# The spatial effect of protein deuteration on nitroxide spin-label relaxation: Implications for EPR distance measurement

**DOI:** 10.1016/j.jmr.2014.09.010

**Published:** 2014-11

**Authors:** Hassane El Mkami, Richard Ward, Andrew Bowman, Tom Owen-Hughes, David G. Norman

**Affiliations:** a School of Physics and Astronomy, University of St. Andrews, St. Andrews KY16 9SS, UK; b Nucleic Acids Structure Research Group, University of Dundee, Dundee DD1 5EH, UK; c Centre for Gene Regulation and Expression, University of Dundee, Dundee DD1 5EH, UK

**Keywords:** EPR, Relaxation, *T_m_*, Spin-label, PELDOR, DEER, Deuteration

## Abstract

Pulsed electron–electron double resonance (PELDOR) coupled with site-directed spin labeling is a powerful technique for the elucidation of protein or nucleic acid, macromolecular structure and interactions. The intrinsic high sensitivity of electron paramagnetic resonance enables measurement on small quantities of bio-macromolecules, however short relaxation times impose a limit on the sensitivity and size of distances that can be measured using this technique. The persistence of the electron spin-echo, in the PELDOR experiment, is one of the most crucial limitations to distance measurement. At a temperature of around 50 K one of the predominant factors affecting persistence of an echo, and as such, the sensitivity and measurable distance between spin labels, is the electron spin echo dephasing time (*T_m_*). It has become normal practice to use deuterated solvents to extend *T_m_* and recently it has been demonstrated that deuteration of the underlying protein significantly extends *T_m_*. Here we examine the spatial effect of segmental deuteration of the underlying protein, and also explore the concentration and temperature dependence of highly deuterated systems.

## Highlights

• Echo decay curves were measured on segmentally deuterated, octameric proteins.
• The echo dephasing time (*T_m_*) was extracted from each data set.
• Spin relaxation was correlated to the spatial distribution of protons.
• Temperature and concentration dependence of *T_m_* and *T*_1_ was measured.
• The effect of deuteration on relaxation and dipolar coupling were discussed.

## Graphical abstract

**Figure:**
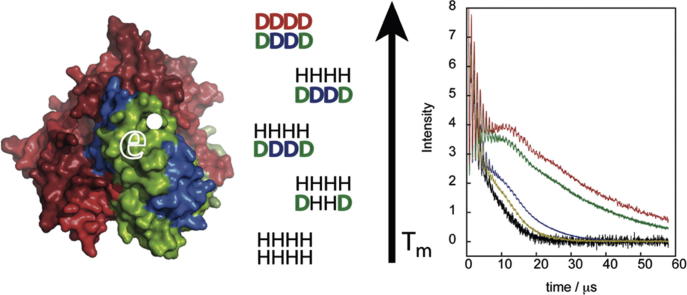

## 1 Introduction

In a non-deuterated environment, short spin echo dephasing times (*T_m_*) [1], [2], [3], in the order of 2–4 μs, are usually observed, when studying nitroxide spin-labeled proteins, in frozen solution at around 50 K. A *T_m_* of 2 μs limits the measurement of distances, in the PELDOR experiment [4], [5], to around 3–4 nm and also limits the sensitivity. *T_m_* is affected by contributions from instantaneous and spectral diffusion as well as hyperfine interactions with surrounding nuclei. Unpaired electrons can show dipolar coupling to nuclear spins in the surrounding media and although individual nuclear spin flip is slow, the large number of coupled nuclei in a typical protein makes these events highly probable and spin flips in dipolar coupled nuclei change the precession frequency of the unpaired electron. Dipolar coupling is proportional to the magnetic moment, so proton spin diffusion is a more effective mechanism of dephasing electron spins than would be deuterium [6] and as a result the use of deuterated solvents can moderately increase the *T_m_* to around 5–6 μs [1]. More significantly, it has been demonstrated that total deuteration of a protein, containing a site-specific nitroxide spin-label pair extended the *T_m_* dramatically, giving a value of approximately 36 μs [7]. A *T_m_* of this magnitude permits substantial increase in the maximum distance measurement, better background correction, more accurate distance distribution determination and considerably higher sensitivity. Although total system deuteration has demonstrated dramatic increases in *T_m_*, no study has previously investigated the detailed spatial relationship between protein deuteration and *T_m_* or indeed examined the temperature and concentration dependence of relaxation under these conditions. The relaxation time *T_m_* can be described by an equation utilizing a homogeneous concentration of protons around the spin label [8], [9]. This model is suitable to describe relaxation caused by the solvent but is inadequate in its description of relaxation caused by the structured environment of the underlying protein. The exact details of spin diffusion in complicated spin-labeled biological systems are poorly understood but appear to be related to the size of the electron–nuclear and nuclear–nuclear couplings, the chemical and dynamic nature of the surrounding environment and the spatial distribution of nuclear spins [3]. Here we report the effects of segmental protein deuteration on observed *T_m_*, providing a unique observation of the spatial relationship between the spin-labels and protein protons and the extent and impact of spin diffusion.

The histone core octamer is composed of two copies of each, H3, H4, H2A and H2B histones, in a spool or bobbin like structure made up of a central H3/H4 tetramer sandwiched between two H2A/H2B dimers. Previous work demonstrated the measurement of a large number of distances between nitroxide spin labels situated on either the H3 or the H4 histones within the intact histone octamer [10]. The octameric nature of this protein complex allows assembly with either all, a subset, or none of the histones deuterated. Segmentally deuterated core octamer allows investigation of the effect of spatial distribution, of protein protons on *T_m_* and other relaxation pathways.

**Fig. 1:**
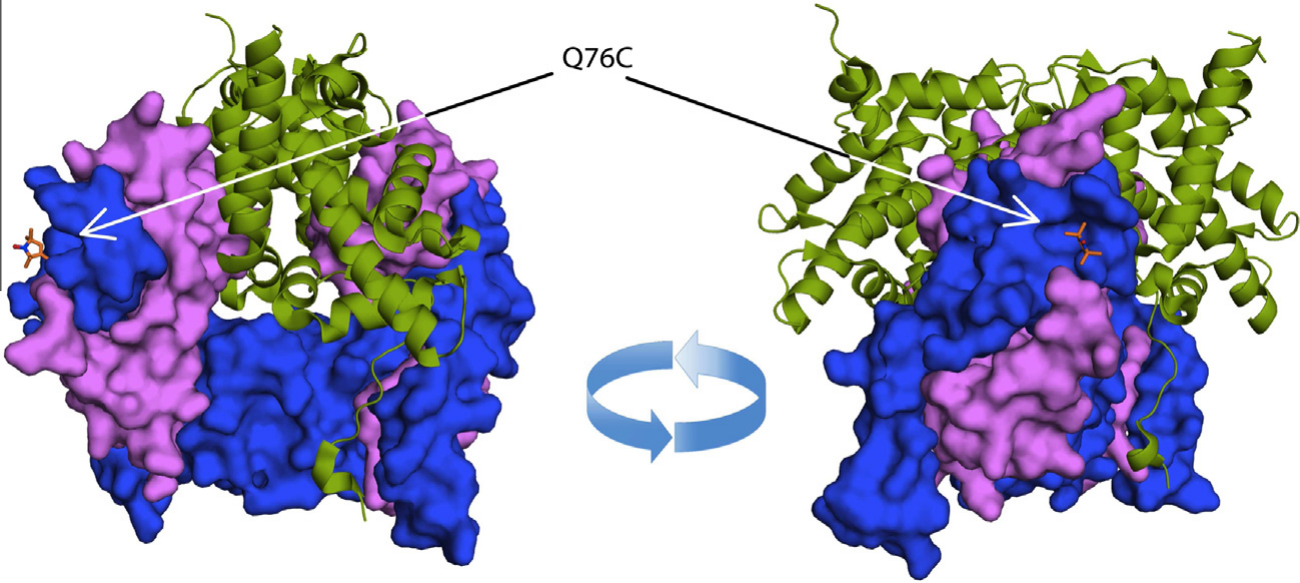
Structure of the modified histone octamer (PDB code 1TZY). Two rotated views showing the position of the spin label at Q76C on histone H3 (blue surface), Histone H4 shown by magenta surface and H2A–H2B shown by green cartoon. (For interpretation of the references to colour in this figure legend, the reader is referred to the web version of this article.)

We have derivatized the ‘nucleosome core octamer’ using MTSSL (Fig. 1) at the mutated position Q76C of histone H3, thus generating a symmetrical pair of label sites within the octamer, with a spin-label distance of 70 Å [11]. Measurements of *T_m_* were made on histone octamer constructs in which (i) no histones were deuterated (non-D), (ii) H3 histones were deuterated (H3-D), (iii) H3 and H4 histones were deuterated (H3-D/H4-D), (iv) H4 histones were deuterated, (v) all histones were deuterated (All-D). In this context deuteration specifically refers to the non-freely-exchanging protons of the proteins. Because experiments are conducted in deuterated aqueous buffer, all freely exchanging protons are expected to be in the deutero form.

## 2 Experimental

### 2.1 Sample preparation

The preparation of histones and the assembly of the nucleosome core octamer was essentially as previously described [10], [12]. Briefly, protein expression was achieved using bacterial expression in Rosetta 2 cells (Stratagene) from pET3d expression vectors (peptide sequences shown in Fig. S1). Histone H3 contained the mutations C110A, to remove an unwanted labeling site, and Q76C to introduce the desired labeling site. Freshly transformed cells were grown to stationary phase in 4 ml of 2YT media containing ampicillin and chloramphenicol for selection. For deuteration cells were pelleted, washed once with deuterated media (Spectra9, Cambridge Isoptope Laboratories Inc.), pelleted again and used to inoculate a 250 ml culture in deuterated media. Protein expression was induced by the addition of 1 mM IPTG when the optical density at 600 nm reached 0.6. Induction was carried out at 37 °C for 14 h. Cultures were spun down and re-suspended in 2 ml of Wash Buffer (100 mM NaCl, 20 mM HEPES-KOH pH 7.5, 1 mM EDTA, 1% Triton X-100, 1 mM DTT) and lysed by sonication. Histones were present in the insoluble fraction, which was further washed once in wash buffer and twice in wash buffer without Triton X-100. The insoluble histones were re-dissolved in 4 ml of unfolding Buffer (7 M Guanidinium-HCl, 20 mM HEPES-KOH pH 7.5, 1 mM EDTA, 1 mM DTT) and dialysed into SAU200 Buffer (20 mM sodium acetate pH 5.2, 7 M urea, 200 mM NaCl, 1 mM EDTA, 5 mM β-mercaptoethanol). 0.5 ml of cation exchange resin (SP FF, GE Healthcare) was equilibrated with SAU200 buffer in 10 mL disposable chromatography columns (Bio-Rad). Dialysed histones were bound to the resin, washed twice with 2 mL of SAU200, once with 2 mL of SAU400 (400 mM NaCl), and eluted in 2 mL of SAU800 (800 mM NaCl). Eluted histones were dialysed into H_2_O plus 5 mM β-mercaptoethanol and lyophilized.

Histones were re-dissolved in unfolding Buffer, quantified by absorbance at 280 nm and mixed in equimolar amounts. The octamer complex was refolded by dialysis into refolding buffer (2 M NaCl, 20 mM HEPES-KOH pH 7.5, 1 mM EDTA, 5 mM β-mercaptoethanol), and purified from mis-folded aggregates by gel filtration on a GL 10/300 column packed with Superdex S200(GE Healthcare). Before gel filtration, 20 mM dithiothrietol was added to the samples and incubated at 25 °C for 30 min to ensure complete reduction of the histone H3 labeling site. Gel filtration was carried out in Refolding Buffer without β-mercaptoethanol. Immediately after gel filtration, fractions containing the correctly folded histone octamer were concentrated, using an Amicon Ultra-4 centrifugal concentrator (Millipore) with a molecular weight cut off of 10,000 Da, to ∼25 μM, and spin labeled with a ten-fold excess of non-deuterated (1-Oxyl-2,2,5,5-tetramethylpyrroline-3-methyl) methanethiosulfonate (MTSSL) (Fig. S2) at 25 °C for 3 h.

Excess MTSSL was removed by dialysis verses 2 L of refolding buffer without reducing agents at 4 °C for 16 h. Labeled octamer was combined with a 1-fold excess of H2A–H2B dimers, refolded and purified separately, as our previous work had shown that an excess of dimer stabilizes the octamer complex [10]. H_2_O in the samples was exchanged for D_2_O by four rounds of sequential concentration and dilution, with deuterated refolding buffer minus reducing agent (prepared by lyophilisation and re-solvation of buffer with D_2_O), using Amicon Ultra-4 centrifugal concentrators (Millipore), achieving 99.8% exchange with D_2_O. The octamer samples were finally concentrated to 50 μM and diluted 1:1 with D8-glycerol (Cambridge Isotope Laboratories Inc.), giving a final spin-pair concentration of approximately 25 μM, and stored at 4 °C until EPR measurements were made. Solvent exchange and subsequent sample preparation steps took approximately 2.5 h at room temperature and subsequently samples were routinely stored at 4 °C for several days. Based on reported hydrogen–deuterium exchange rates in proteins [13] and the inherent structural lability of the core histone octamer, it was expected that almost complete exchange of protons would have been achieved.

The extent of deuteration was estimated by mass spectrometry, as previously described [7].

### 2.2 EPR

All experiments were carried out using a Bruker ELEXSYS E580 spectrometer operating at X-band with a dielectric ring resonator 4118X-MD4 and a Bruker 400U microwave source unit. All measurements were made at 50 K with the sample in a frozen glassy state. The resonator was over-coupled giving a Q factor of approximately 100. The video bandwidth was set to 20 MHz.

Experiments to determine the phase memory time (*T_m_*) were performed by measuring the intensity of a Hahn echo as it decayed with increasing inter-pulse delay. The pulse sequence used was *π*/2–t1–*π*, where the *π* pulse was 32 ns and the initial time delay t1 was 400 ns, in addition two-step phase cycling was employed to eliminate receiver offsets. Timings and delays were used appropriately for each sample. The experiment repetition time was 4 ms and 50 shots were taken at each time point.

Echo decay curves in a deuterated medium are dominated, initially, by ESEEM oscillations and so *T_m_* was estimated by fitting of Eq. (1) to the tail end of the data that is largely free of ESEEM.

### 2.3 Calculation of spatial relationships between spin label and protons

Histone octamers used to make relaxation measurements were full length and as such contained unstructured tails that are not defined by X-ray crystallography. In order to calculate the sum(1/*r*^3^) values depicted in Fig. 3, the unstructured tails were built onto the crystal structure (PDB code 1TZY) using a simulated annealing protocol within Xplor-NIH [14]. High temperature dynamics with only the unstructured tail regions allowed to move freely, gave a large ensemble of structures. The position of the spin-label was determined my molecular dynamics as previously described [15]. Distances between the nitrogen atom (averaged position) of the spin label to the positions of any remaining (after deuteration) proton positions were measured and their 1/*r*^3^ values were averaged and summed to provide a single term describing the proton distribution around the unpaired electron and the protons of the protein.

## 3 Results

**Fig. 2:**
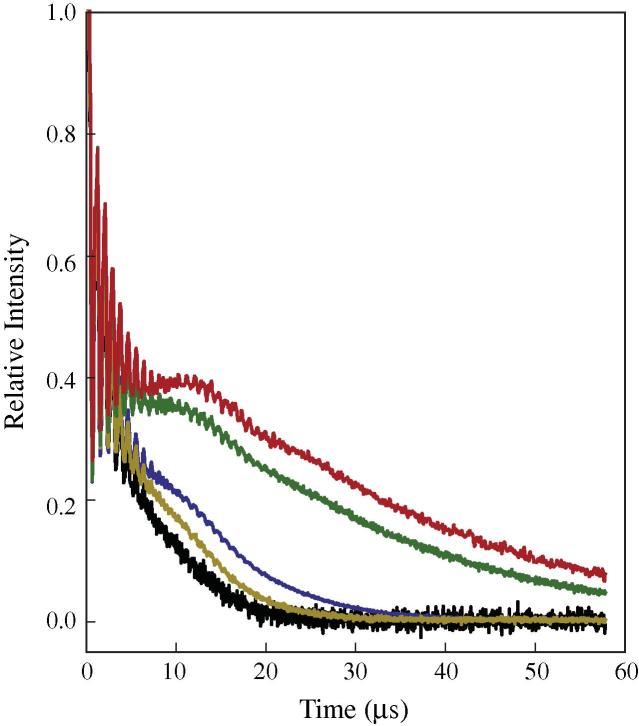
Echo decay curves for segmentally deuterated, spin labeled histone core octamer. Non-deuterated (black), H4 deuterated (yellow), H3 deuterated (blue), H3H4 deuterated (green), fully deuterated (red). Fitted curves are shown in S3. (For interpretation of the references to colour in this figure legend, the reader is referred to the web version of this article.)

Echo decay curves were measured and Fig. 2 shows the complete echo decay curves for all protein constructs discussed here. Echo decay curves were fitted using a stretched exponential (Eq. (1)) [3]. At X-band the beginning of the decay curves are obscured by deuterium ESEEM signals and so fitting and extraction of *T_m_* values was done using cropped decay curves (Fig. S3). Line fitting was also hindered by the presence of a low frequency oscillation, derived from dipolar coupling, that was especially prominent in the fully deuterated sample [16]. The estimated *T_m_* for the non-deuterated, octameric complex (in deuterated solvent) is 6.9 μs, which is at the high end of reported *T_m_* values for a spin label situated on the surface of a protein dissolved in deuterated buffer [1]. Deuteration of H3 leads to an approximate doubling of the *T_m_* to 13.6 μs. The spin-labels, situated on the histone H3, place them quite close to parts of histone H4 and the effect of deuterating only histone H4 also has a large effect, raising the *T_m_* to 11.6 μs, however the combined effect of deuterating both H3 and H4 leads to an even larger increase in *T_m_* to 31 μs. The histone core octamer is structurally divided into two parts, one being the H3/H4 tetramer and the other being made up of a pair of histone H2A/H2B dimers. Deuteration of all histones in the octamer resulted in a final *T_m_* value of 36 μs. This final increase in *T_m_* on deuteration of the H2A/H2B histones is perhaps the most surprising, as the closest part of H2A or H2B to the spin label on H3 is about 20 Å. *T_m_* values were estimated by fitting the experimental echo decay data to a stretched exponential (Eq. (1)) and are listed in Table 1.

(1) $$Y(\tau )=y_{0}\exp\left[-{\left(\frac{\tau }{T_{m}}\right)}^{x}\right]$$

The relationship between the spatial distribution of protons, deuterons and spin-labels is undoubtedly complex. The individual interaction between electron and proton is proportional to the inverse of the distance to the power 3, however if we plot this relationship between distance and *T_m_*, as observed in this system, we see that although a relationship exists, it is not linear. The interaction between electrons, protons and deuterons is clearly influenced by the spatial distribution of interacting species.

**Fig. 3:**
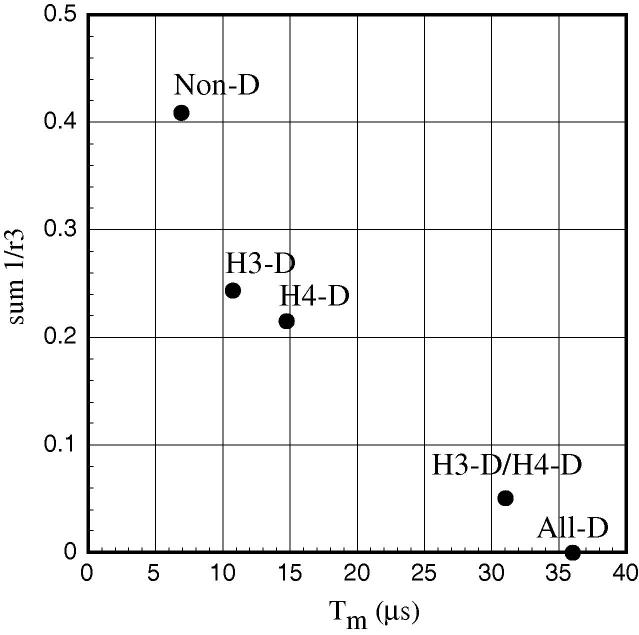
The observed relationship between *T_m_* and sum(1/*r*^3^) for the five partially or completely deuterated histone constructs.

**Table 1:** The estimated values for *T_m_*, *x* (the exponent to the stretched exponential as shown in Eq. (1) and the sum(1/*r*^3^) value describing the spatial distribution of protons around the spin-label. Derived for the segmentally deuterated spin-labeled histone core otamer.

Construct	*T_m_* (μs)	*x*	Sum(1/*r*^3^)
Non-D	6.9	1.6	0.4088
H3-D	14.7	1.5	0.2441
H4-D	10.7	1.5	0.2156
H3-D/H4-D	31.0	1.3	0.0510
All-D	36.0	1.2	0.0000

**Fig. 4:**
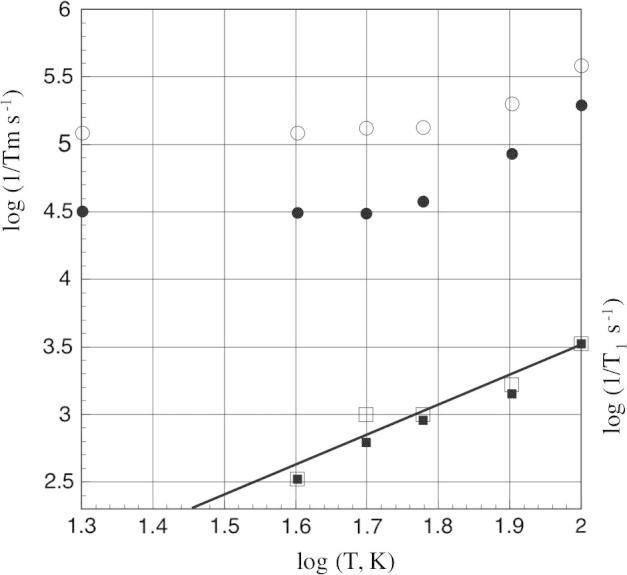
The temperature dependence of log(1/*T*_1_and 1/*T_m_*) for fully and non deuterated histone octamers. Open symbols non-deuterated protein, filled symbols fully deuterated protein. Upper data is log(1/*T_m_*), shown with an interpolated curve. Lower data and best-fit line are log(1/*T*_1_) data (best fit lines are indistinguishable).

The temperature dependence of the electron spin longitudinal relaxation rate, 1/*T*_1_, and the rate constant of the echo dephasing, 1/*T_m_*, for non-deuterated and all-deuterated histone octamers are shown in Fig. 4. One can distinguish between two temperature dependence regimes (below and above 50 K). At temperatures <50 K, log(1/*T_m_*) is practically independent of temperature and saturates at 5.1 s^−1^ and 4.5 s^−1^ for Non-D and All-D respectively. The fact that the limiting value of log(1/*T_m_*) is dependent on whether the protein is protonated or deuterated suggests that *T_m_* at low temperature is dominated by the nuclear spin diffusion due to the mutual spin flip-flops [17]. This conclusion is consistent with the results obtained for H3-D, H4-D, H3-D/H4-D and fully deuterated samples (All-D) seeing that the more protons are exchanged with deuterium the slower is the rate of echo dephasing (1/*T_m_*). The slower (1/*T_m_*) rate is because the deuteron has a magnetic moment that is 6.51 times smaller than for protium, which results in a smaller influence on electron spin dephasing. Between 50 and 100 K the phase memory relaxation rate for both samples, Non-D and All-D, increases indicating that a thermally activated process arises. Earlier studies have implicated the rotation of the spin-label methyl groups in this effect [2], [18], [19]. It has been shown that modification of the nitroxide label, eliminating the methyl groups by cyclization, largely eliminated the change in *T_m_* between 50 and 100 K. In this study the spin labels are non-deuterated and contain geminal methyl groups. The temperature dependence of 1/*T_m_* rate yielded an activation energy of 1 kcal/mol, which is comparable to other values obtained for methyl group rotation in several nitroxyls [18]. Perhaps surprisingly, deuteration of the spin label methyl groups appears to have no effect on this temperature transition (data not shown). Thus the most significant gains in *T_m_* due to protein deuteration are only observed at temperatures around 50 K and below.

Unlike the 1/*T_m_* temperature dependence, the spin longitudinal relaxation rate (1/*T*_1_) does not show any major difference between the non-deuterated and all-deuterated samples, which indicates that within this temperature range, the nuclear spins do not play a significant role in the spin–lattice relaxation mechanism. For both samples 1/*T*_1_ shows slight temperature dependence, and during the observed temperature range it does not approach the value of 1/*T_m_* suggesting that *T*_1_ processes do not have a significant effect on the electron spin echo dephasing [3].

We have shown the strong effect of protein deuteration on *T_m_*. However as *T_m_* is extended it becomes more sensitive to other effects like instantaneous diffusion and electron spin–spin diffusion [17]. The electron spin echo dephasing observations, in which the histone octamer was increasingly segmentally deuterated, showed, in addition to strong ESEEM modulations, an oscillation resulting from the electron dipole–dipole interaction between the two spin labels present on the protein (see Fig. 2). Such distance dependent dipolar interactions were only observed in the case of H3-D/H4-D and All-D samples due to their long *T_m_*. In Fig. 2 we can see that the longer the *T_m_*, the more pronounced the electron dipole–dipole interaction. The observation that the 2 pulse ESE experiment is capable of detecting electron spin–spin interactions in biradicals has been made previously [16]. In a two-pulse echo experiment, when the second pulse is applied and flips two dipole-coupled spins simultaneously, the dipole interaction does not refocus and leads to a dephasing of spin pairs, this effect is known as instantaneous diffusion. Two cases can be envisaged. One situation is where the spin pairs are randomly distributed and so there will be a wide distribution of dipolar interactions, *D*, and therefore the echo oscillations, occurring at a range of frequencies, average out, leaving only an exponential-like echo decay contribution. In the second case, when the spin pairs have a defined dipole interaction, *D*, the echo decay will be modulated by the dipole–dipole frequency, *y*(*τ*) ∼ cos(*Dτ*) [16]. The H3-D/H4-D and All-D histone octamer constructs clearly fulfil the requirements for a dipolar interaction to be observed in a 2 pulses ESE experiment since they are double spin labeled, with a defined dipole–dipole interaction, and have long *T_m_*. The Fourier transform of the ESE decay yielded a dipolar coupling which is in good agreement with the PELDOR data (see supplementary material Fig. S4a and b).

**Fig. 5:**
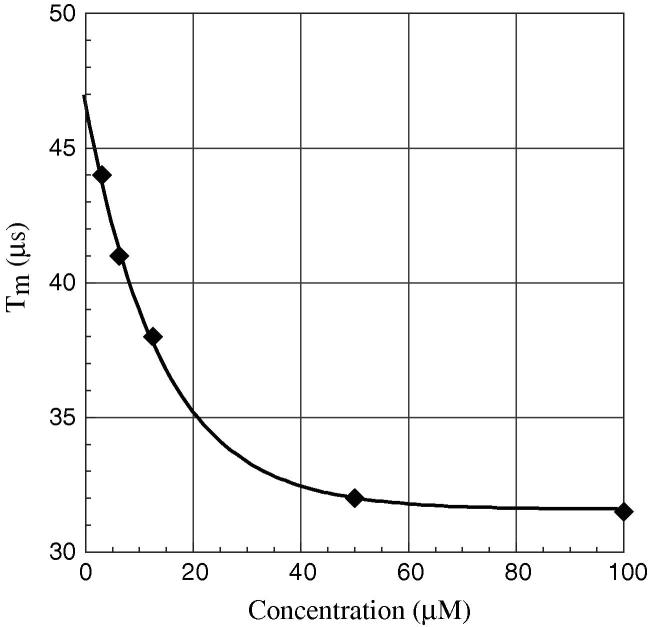
Graph showing the decrease of *T_m_* with increasing concentration of fully deuterated spin-labeled histone octamer.

In order to get more insight into the effect of deuteration, we have also studied the concentration dependence of *T_m_* (Fig. 5) for the fully deuterated sample at 50 K. The data shows that *T_m_* is lengthened by about 30% when the sample is diluted 16 times. This supports our assertion that the nuclear spin diffusion is dominating the echo dephasing at low temperature, given that at the same temperature, we measured an increase of 80% of the *T_m_* while going from non-deuterated to fully deuterated protein. The slight improvement shown in the concentration dependence is probably related to the reduction of the other factors affecting the spin dephasing, such as instantaneous diffusion [20], [2]. It is worthwhile to note that the *T_m_* traces for all concentrations, show the electron dipole–dipole modulation but with larger enhancement at lower concentration.

## 4 Discussion and conclusion

We have demonstrated the impact of partial segmental deuteration on the electron spin relaxation times. The relaxation effects of deuteration are manifest exclusively on the rate of spin dephasing, *T_m_*. Because spin dephasing is multifactorial and complex with regards to the spatial distribution of dephasing nuclei, there is no obvious, simple correlation to be easily extracted from this data.

The relationship between the distribution of segmental deuteration and *T_m_* is illustrated in Fig. 3 and shows a strong, but not quite linear, correlation between *T_m_* and the distance to the remaining proton distances measured as the sum of the inverse, electron–proton, distances cubed. Because of various limitations and uncertainties in the measurements and the analysis of relatively few data points, significant further investigations utilizing alternative protein constructs will be required to clarify and interpret this situation.

However replacing protein protons with deuterons results in an increase in *T_m_* of 5.5 times and it is empirically shown that most of the effect, of deuteration on the rate of spin dephasing, is due to nuclear–electron spin interactions within about 25 Å of the spin label. The observation that deuteration of protein within 25 Å accounts for much of the effect has interesting application to structural studies of protein complexes, in that even deuteration of parts of a complex can lead to significant gains in sensitivity and the distances measurable. The longest distance so far, measured by pulsed EPR is 102 Å, measured in a deuterated protein system [21]. It is possible to extrapolate from the *T_m_* values measured, to predict that longest distances that could be measured by pulsed EPR would be in the region of 125–130 Å, depending somewhat on the required measurement quality.

The removal of proton driven dephasing has allowed us to see the effect of, what we presume to be, electron dipole–dipole effects on dephasing. In this situation the effect of electron dipole–dipole driven dephasing is rather small in comparison, however dropping the concentration of a deuterated spin-labeled dimer from 50 μM to 3 μM still leads to an increase of *T_m_* of 1.4 times. In this particular case, although instantaneous diffusion can be decreased by dilution, the presence of a double label on the protein may also contribute significantly to instantaneous diffusion and could possibly be reduced by the use of a weaker microwave pulses. We have also shown the enhancement of the electron dipole–dipole modulation in the *T_m_* traces with increasing protein deuteration. Although extraction of clean dipole–dipole modulation, from relaxation curves is difficult due to the complexity of the data, it could be speculated that this may be the most sensitive method of distance measurement using pulsed EPR. The *T_m_* measured for free nitroxide spin label (TEMPONE) in a deuterated matrix, using small pulse turning angles, has been reported as >100 μs [1]. The measurement of *T_m_* from TEMPONE, in deuterated matrix, gave an increase in *T_m_* over that in a protonated matrix of a factor of >25. Even extrapolating our measurements to zero concentration we only get a *T_m_* value of 47 μs, in a double nitroxide spin labeled deuterated protein. Although the experiments described here and the data shown in Fig. 5 are suggestive of instantaneous diffusion it is interesting to speculate as to how much of the missing *T_m_* advantage (over that of TEMPONE) is from the instantaneous diffusion and how much may be from other relaxation routes.
