# Supplementary material for: The spatial effect of protein deuteration on nitroxide spin-label relaxation: Implications for EPR distance measurement
Source: J Magn Reson. 2014 Nov;248:36–41. doi: 10.1016/j.jmr.2014.09.010 (PMC4245719; doi:10.1016/j.jmr.2014.09.010)

Supporting Information

The Spatial Effect of Protein Deuteration on Nitroxide Spin-label Relaxation: Implications to EPR Distance Measurement

Hassane. El Mkami, Richard Ward, Andrew Bowman , Tom Owen-Hughes, David G. Norman

**Contents:**

Sequences of histones S1

Spin label structure S2

Graph showing line fits to decay data S3

Pake patterns derived from PELDOR and FT of ESE decay S4

*S1 Protein sequences used in this study*

H3Q76R1 C110A

ARTKQTARKSTGGKAPRKQLATKAARKSAPATGGVKKPHRYRPGTVALREIRRYQKSTELLIRKLPFQRLVREIACDFKTDLRFQSSAVMALQEASEAYLVALFEDTNLAAIHAKRVTIMPKDIQLARRIRGERA

H4

SGRGKGGKGLGKGGAKRHRKVLRDNIQGITKPAIRRLARRGGVKRISGLIYEETRGVLKVFLENVIRDAVTYTEHAKRKTVTAMDVVYALKRQGRTLYGFGG

H2A

SGRGKQGGKTRAKAKTRSSRAGLQFPVGRVHRLLRKGNYAERVGAGAPVYLAAVLEYLTAEILELAGNAARDNKKTRIIPRHLQLAVRNDEELNKLLGRVTIAQGGVLPNIQSVLLPKKTESSKSAKSK

H2B

AKSAPAPKKGSKKAVTKTQKKDGKKRRKTRKESYAIYVYKVLKQVHPDTGISSKAMSIMNSFVNDVFERIAGEASRLAHYNKRSTITSREIQTAVRLLLPGELAKHAVSEGTKAVTKYTSAK

S2 Figure showing the structure of the MTSSL spin-label used in this study


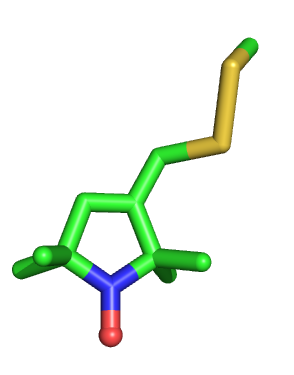


S3 Graph showing fitted lines to data using equation 1. Non-deuterated (black), H4-deuterated (red), H3-deuterated (purple), H3/H4=deuterated (blue), all deuterated (green).


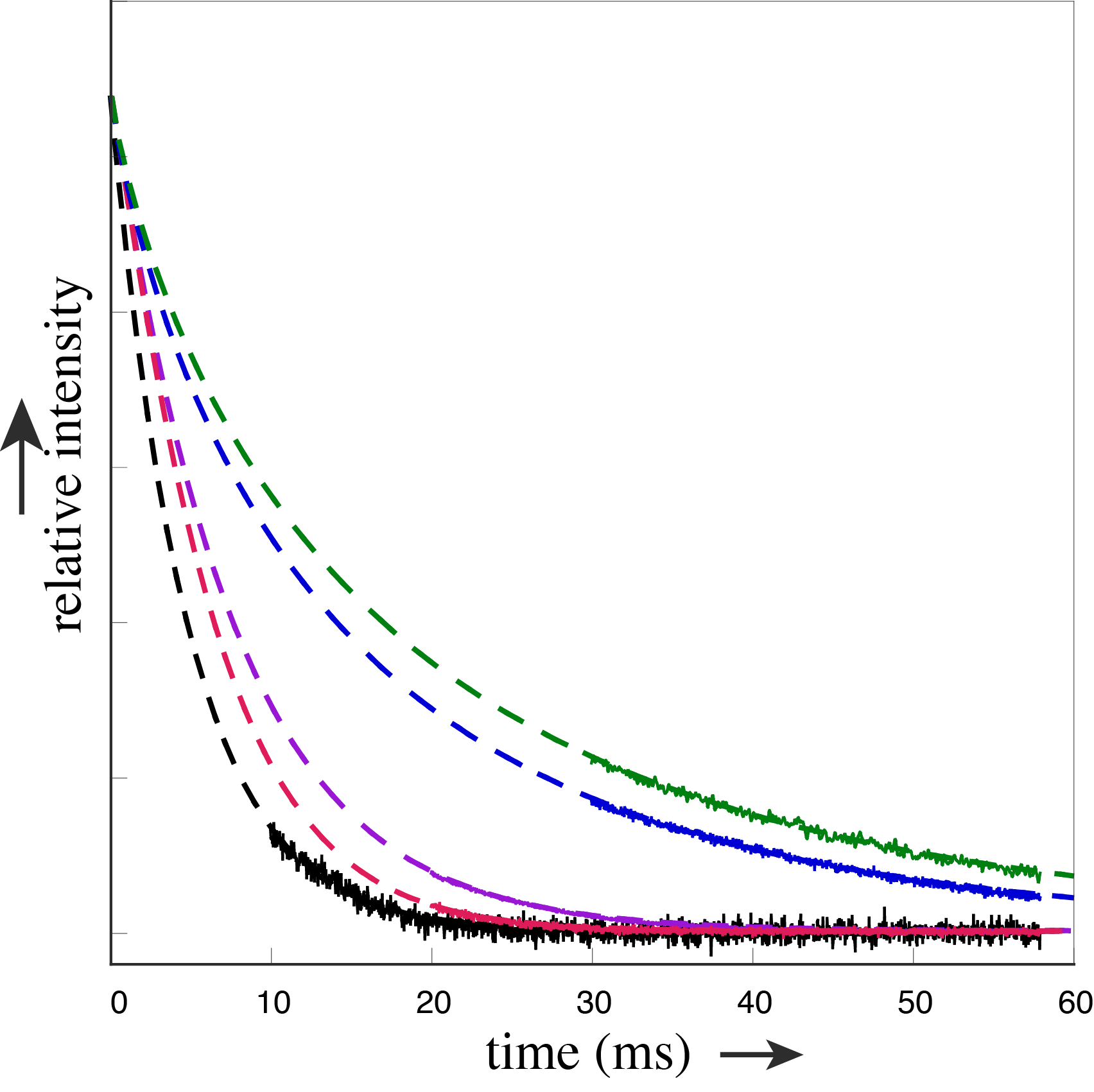


*S4 Pake pattern obtained from Peldor (blue) and FT of ESE decay (red). (A) full comparison, including ESEEM signal (B) expanded region showing dipolar derived signal only.*


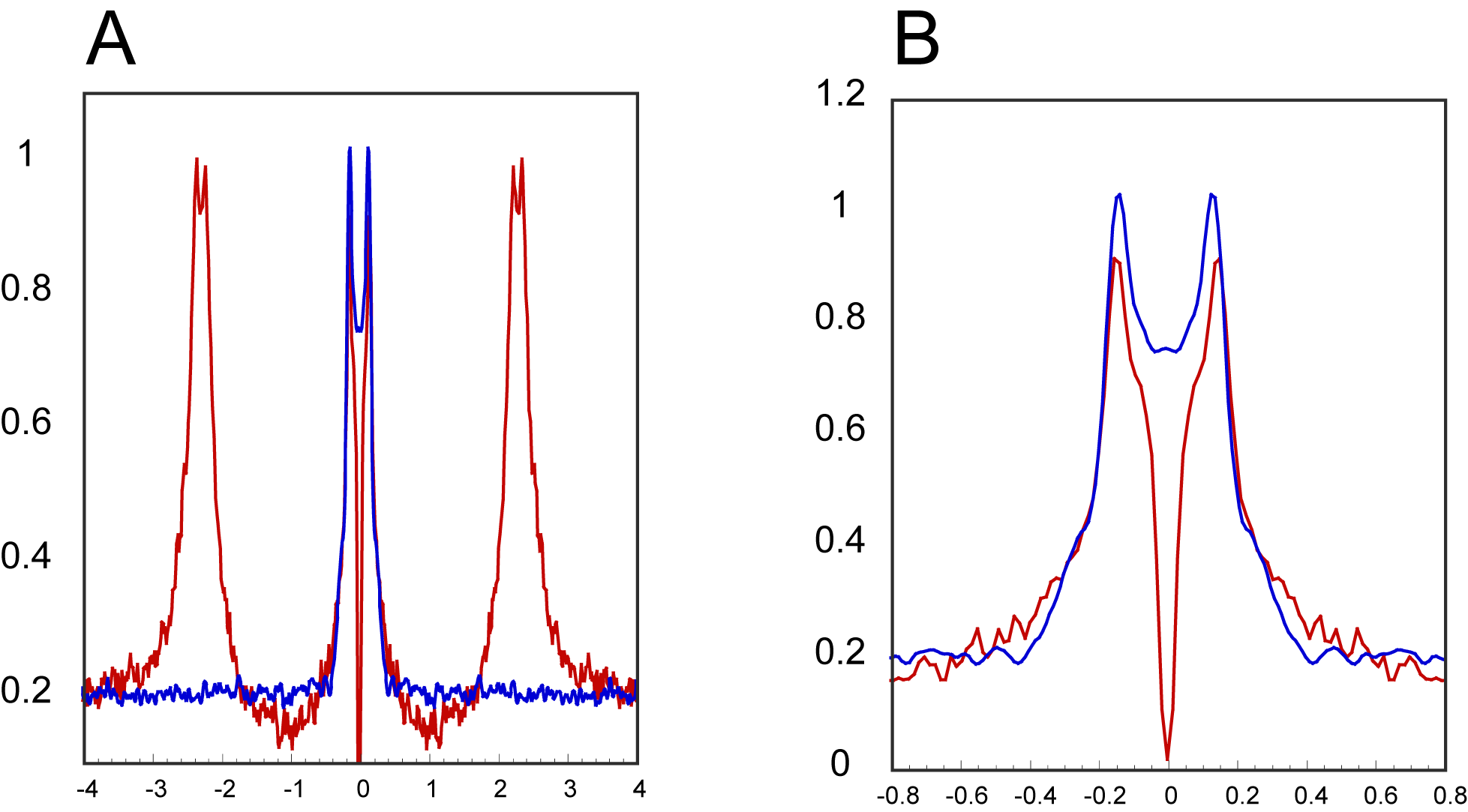

Supplement: Supplementary data 1 [file mmc1.docx]
